# The Digestive Function of *Pseudoplatystoma punctifer* Early Juveniles Is Differentially Modulated by Dietary Protein, Lipid and Carbohydrate Content and Their Ratios

**DOI:** 10.3390/ani11020369

**Published:** 2021-02-02

**Authors:** Diana Castro-Ruiz, Karl B. Andree, Mikhail M. Solovyev, Christian Fernández-Méndez, Carmen García-Dávila, Chantal Cahu, Enric Gisbert, Maria J. Darias

**Affiliations:** 1 Laboratorio de Biología y Genética Molecular (LBGM), Dirección de Investigación en Ecosistemas Acuáticos Amazónicos (AQUAREC), Instituto de Investigaciones de la Amazonía Peruana (IIAP), Carretera Iquitos-Nauta km 4.5, Iquitos, Peru; cgarcia@iiap.gob.pe; 2 Aquaculture Program, Centre de Sant Carles de la Ràpita (IRTA-SCR), Institut de Recerca i Tecnologìa Agroalimentaries (IRTA), Crta. Poble Nou km 5.5, 43540 Sant Carles de la Ràpita, Spain; karl.Andree@irta.es (K.B.A.); Enric.Gisbert@irta.cat (E.G.); 3 Institute of Systematics and Ecology of Animals Siberian Branch, Russian Academy of Sciences, 11 Frunze St., 630091 Novosibirsk, Russia; yarmak85@mail.ru; 4 Tomsk State University, 36 Lenin Ave., 634050 Tomsk, Russia; 5 Laboratorio de Bromatología, Dirección de Investigación en Ecosistemas Acuáticos Amazónicos (AQUAREC), Instituto de Investigaciones de la Amazonía Peruana (IIAP), Carretera Iquitos-Nauta km. 4.5, Iquitos, Peru; cfernandez@iiap.gob.pe; 6 LEMAR, Univ Bretagne, CNRS, Ifremer, IRD, 29280 Plouzané, France; ccahu@ifremer.fr; 7 MARBEC, Univ Montpellier, CNRS, Ifremer, IRD, Montpellier, France

**Keywords:** neotropical fish, aquaculture, digestive enzymes, gene expression, nutrition, juveniles, histology, development, macronutrients, diet

## Abstract

**Simple Summary:**

The Neotropical catfish *Pseudoplatystoma punctifer* is a promising candidate species for aquaculture diversification in the Amazon basin. However, optimized feeding strategies are needed to improve growth and survival during the early life stages. In order to determine the nutritional needs of early juveniles, the influence of four diets containing different protein and lipid content in their digestive physiology and performance was evaluated. To do so, the gene expression and the activity of the main digestive enzymes, the histology of the digestive organs, and the incorporation of the dietary macronutrients in the body tissue of fish fed the different diets were analyzed. The diet containing the highest protein and lipid content promoted the fastest development of the digestive capacities, resulting in a better growth and survival that increased sixfold and twofold, respectively, in comparison to previous feeding protocols. Lipids were favored over carbohydrates as a source of energy and excessive carbohydrate content led to unbalanced lipid metabolism and fat deposition in the liver. This study provided new knowledge on the molecular and biochemical mechanisms underlying nutrient digestion in this species and on the nutritional needs of early juveniles, which represents a significant contribution towards the establishment of its commercial culture.

**Abstract:**

*Pseudoplatystoma punctifer* is an Amazonian catfish highly appreciated for its high flesh quality, size, and commercial value. Its aquaculture is pursued to satisfy the demands of an increasing population in the region. However, knowledge of the nutritional needs during the early life stages is necessary for improving growth and reducing the incidence of cannibalism, factors that limit the success of its commercial farming. This study aimed at evaluating the influence of four diets containing different protein and lipid levels (30:15, 30:10, 45:15, or 45:10 in %) in the digestive physiology and performance of early juveniles. The results showed that the dietary protein:lipid as well as carbohydrate levels and ratios influenced differently the whole-body proximate composition, the digestive physiology and development, and hence growth and survival. The 45:15 diet promoted the best growth, survival, and the most rapid development of the digestive system, as shown at histological (higher number of hepatocytes, goblet cells in the anterior intestine and enterocytes in all intestinal portions, and longer folds in the posterior intestine), molecular (highest *amylase*, *lipoprotein lipase*, *phospholipase*, *trypsinogen,* and *pepsinogen* gene expression), and biochemical (highest lipase and pepsin activities and higher alkaline phosphatase:leucine alanine peptidase activity ratio) levels. Lipids were favored over carbohydrates as source of energy, with lipids promoting a protein-sparing effect at adequate energy:protein ratio. Carbohydrate content higher than 25% was excessive for this species, leading to unbalanced lipid metabolism and fat deposition in the liver.

## 1. Introduction

In fish farming, a balanced diet in terms of macro- and micronutrients is essential to assure survival, as well as optimal development, health, and growth, especially during the early life stages, when organs and systems are developing. Nutritional requirements are specific for each species, vary throughout development and depend on rearing conditions. Therefore, this knowledge is key to formulate feeds adapted to these specific dietary needs. 

Feed costs usually represent the highest operating expense in aquaculture, with dietary proteins being the main and most expensive component of fish feeds. In order to spare proteins from energy metabolism, and hence reduce production costs, research efforts have been largely focused on replacing part of the protein content with nonprotein energy sources. However, a balanced dietary energy:protein (E:P) ratio is necessary for the regulation of food intake and metabolism of nutrients, including carbohydrates and lipids [1,2,3]. When nonprotein energy is not adequate and/or not supplied in sufficient amounts, proteins can be catabolized to meet the energy requirements in detriment of growth [4]. The most efficient diets contain both lipids and carbohydrates in adequate composition and amount allowing to spare protein to meet the global energy requirements and to provide proteins for somatic growth. The composition and content of these energy sources have a direct effect on growth, feed conversion efficiencies, nutrient retention, and body composition [1,2,5,6,7,8,9,10,11,12,13]. Lipids are the most preferred energy source for many fish species, especially the carnivorous ones. However, excessive lipid content may lead to reduced growth and increased body lipid deposition [6]. In general, carbohydrates are not the principal source of energy or carbon for fish and are poorly utilized by most carnivorous fish [5,14], although they can improve protein utilization in some species [8,15]. Since carbohydrates are less expensive than lipids, an increase in the level of inclusion in diets is sought, especially in herbivorous and omnivorous species that use starch more efficiently than carnivorous fish [14,16]. As with lipids, an excessive dietary carbohydrate content can alter the energy metabolism, leading to fat body deposition, and ultimately impairing growth [17,18]. Therefore, the optimum dietary ratios between protein and energy as well as between carbohydrate and lipid in fish feeds need to be carefully defined in order to promote a healthy development and an optimal growth and survival. 

*Pseudoplatystoma punctifer* is a piscivorous migratory catfish native to the Amazon basin in Bolivia, Brazil, Colombia, Ecuador, Peru, and Venezuela [19]. Given its high flesh quality, size (up to 1.40 m total length, TL), and commercial value, *P. punctifer* is highly appreciated in the region and suffers from high fishing pressure [20,21], what has led to the development of the aquaculture of this species. However, a high incidence of cannibalism and a low acceptability of compound diets at weaning have been responsible for low survival during early life stages [22,23], hence limiting the success of its commercial farming. Recent studies on the morphological and functional ontogeny of the digestive system of *P. punctifer* [23,24] have shown that the transition from the larval to the juvenile stage from a digestive physiology point of view (transition from alkaline to acid digestion) occurs at around 10–12 days post fertilization (dpf; 12.02 ± 0.18 mm TL) at 28 °C, allowing to successfully wean individuals 1 weak earlier in comparison to previous rearing protocols [25]. In Darias et al. [25], we studied the nutritional needs of *P. punctifer* during the early juvenile stage (from 13 to 26 dpf) using four compound diets containing different protein:lipid (P:L) levels (30:15, 30:10, 45:15, or 45:10, in %). Low protein diets were formulated to evaluate the potential protein-sparing effect of other energy sources, such as lipids and carbohydrates. The highest growth performance and lowest incidence of cannibalism was observed in individuals fed the 45:15 diet, where the increase in energy from lipids resulted in sparing proteins for growth [25].

With the aim to deepen in the understanding of the nutritional physiology of *P. punctifer* during early life stages, in this paper, we focused on the effect of these diets on the development and function of the digestive system of early juveniles of *P. punctifer* at histological, molecular, and biochemical levels. Regarding the latter, the study focused on the expression of the main digestive genes (trypsinogen (*try*), chymotrypsinogen (*chy*), amylase (*amy*), lipoprotein lipase (*lpl*), phospholipase (*phl*), pepsinogen (*pep*)) and the activity of intestinal (alkaline phosphatase—AP and leucine alanine peptidase—LAP), pancreatic (α-amylase, bile salt-activated lipase, trypsin, and chymotrypsin) and gastric (pepsin) enzymes.

## 2. Materials and Methods 

### 2.1. Rearing and Feeding Protocol

Larvae were obtained by hormonally induced spawning of a sexually mature couple of *P. punctifer* (♀: 3.6 kg; ♂: 1.85 kg) from a broodstock maintained in captivity at the Instituto de Investigaciones de la Amazonia Peruana (IIAP, Iquitos, Peru). The female and male were injected intramuscularly with carp pituitary extract (Argent Chemical Laboratories, Inc., Redmond, WA, USA) at 5 mg kg^−1^ and 1 mg kg^−1^ of body weight, respectively, according to Darias et al. [25]. Fertilized eggs (fertilization rate ~99.9%) were incubated at 28 °C in 60 L cylindroconical tanks connected to a freshwater recirculating system. Hatching took place 20 ± 2 h later (hatching rate ~96%) and larvae were transferred at 3 dpf into a 30 L tanks connected to a clear water recirculation system with mechanical and biological filters. Water conditions were: temperature: 27.8 ± 0.7 °C, pH: 7.0 ± 0.5, dissolved oxygen: 7.4 ± 0.2 mg L^−1^, NO_2_^−^: 0.38 ± 0.27 mg L^−1^, NH_4_^+^: 0.26 ± 0.13 mg L^−1^, and water flow rate: 0.2 L min^−1^. Larvae and early juveniles were reared in triplicate (initial density 30 larvae L^−1^) under a photoperiod of 0L:24D and fed 5 times a day with *Artemia* spp. nauplii from 4 to 12 dpf and weaned from 13 dpf within 3 days onto four experimental compound diets containing different P:L levels (30:15, 30:10, 45:15, or 45:10, Table 1). From 16 dpf, early juveniles were fed only these diets until 26 dpf. Once weaned, individuals were fed five times a day at 5% of the fish wet weight.

### 2.2. Growth and Survival Measurements

The methodology used for growth and survival measurements is described in Darias et al. [25]. In brief, individuals of *P. punctifer* were collected at 12, 20, and 26 dpf and euthanized with an overdose of Eugenol (0.05 μL mL^−1^; Moyco^®^, Moyco, Lima, Peru). In order to monitor growth, 15 individuals were placed in a Petri dish, photographed using a scale bar and TL was measured on the digital images (300 dpi) using ImageJ software [26]. Wet weight (WW) was determined using an analytic microbalance (Sartorius BP 211 D, Data Weighing Systems, Inc., Elk Grove, IL, USA, ± 0.01 mg). Survival was calculated at 12 and 26 dpf with respect to the number of individuals at the beginning of each feeding period and calculated considering the number of individuals sampled at each sampling point [25].

### 2.3. Proximate Composition and Fatty Acid Analyses

Sampled individuals of 26 dpf were washed with distilled water at −80 °C until analysis. Total lipids of the compound diets and *P. punctifer* individuals were extracted in chloroform:methanol (2:1, *v/v*) using the method of Folch et al. [27] and quantified gravimetrically after evaporation of the solvent under a nitrogen flow followed by overnight vacuum desiccation. Total lipids were stored in chloroform:methanol (2:1, 20 mg mL^−1^) containing 0.01% butylated hydroxytoluene (BHT) at −20 °C until analysis. Acid-catalyzed transmethylation was carried out using the method of Christie [28]. Methyl esters were extracted twice using isohexane:diethyl ether (1:1, *v/v*), purified on thin-layer chromatography plates (Silica gel 60, VWR, Lutterworth, UK), and analyzed by gas–liquid chromatography on a Thermo Electron Trace GC (Winsford, UK) instrument fitted with a BPX70 capillary column (30 × 0.25 mm id; SGE, Milton Keynes, UK), using a two-stage thermal gradient from 50 °C (injection temperature) to 150 °C after ramping at 40 °C min^−1^ and holding at 250 °C after ramping at 2 °C min^−1^, helium (1.2 mL min^−1^ constant flow rate) as the carrier gas and on-column injection, and flame ionization detection at 250 °C. Peaks of each fatty acid were identified by comparison with known standards (Supelco Inc., Bellefonte, PA, USA) and a well characterized fish oil, and quantified by means of the response factor to the internal standard, 21:0 fatty acid, added prior to transmethylation, using a Chrom-Card for Windows (Trace GC, Thermo Finnigan, Milan, Italy). Results of fatty acid content were expressed as a percentage of total fatty acids (TFA). Protein and carbohydrate contents were determined following the Lowry et al. [29] and the DuBois et al. [30] methods, respectively.

### 2.4. Histological Analyses

Individuals of *P. punctifer* (*n* = 10) were sampled at 26 dpf from each tank and fixed in buffered formaldehyde (pH = 7.2) at 4 °C overnight. The day after, individuals were dehydrated with graded series of ethanol and stored in 70% ethanol at 4 °C until further processing. After the dehydration process, individuals were embedded in paraffin with an automatic tissue processor Histolab ZX-60 Myr (Especialidades Médicas MYR SL, Tarragona, Spain). Then, paraffin blocks were prepared in an AP280-2Myr station and cut into serial sagittal sections (3 μm thick) with an automatic microtome Microm HM (Leica Microsystems Nussloch GmbH, Nussloch, Germany). Paraffin cuts were kept at 40 °C overnight. After that, samples were deparaffinized with a graded series of xylene substitute and stained by means of Periodic Acid Schiff (PAS) and Alcian Blue (AB) at pH 2.5 [31]. Histological preparations were observed under a Leica DM2000 LED microscope equipped with a camera Leica MC170 HD (Leica Microsystems Nussloch GmbH, Nussloch, Germany). The number of (i) mucosa folds and goblet cells of the anterior (AI), middle (MI), and posterior (PI) intestine; (ii) lipid vacuoles in the liver and intestine; and (iii) hepatocytes were counted in 6 randomly chosen fields per specimen (100 µm or 100 µm^2^ per field, depending on the case). In addition, the size of (i) mucosa folds and goblet cells of the AI, MI, and PI regions, (ii) epithelium of the intestine, and (iii) lipid droplets in the liver and intestine were measured (in µm or µm^2^ depending on the case) in 6 randomly chosen fields per specimen. The surface of lipid droplets was calculated on a total of 40 lipid droplets from five fish per sampling point and tank following the formula S = ¼ π a b; where a and b were the minimum and maximum diameters of the item measured [32]. Measurements on histological slides were performed with the ImageJ software and data expressed as the range comprised between the minimum and maximum values recorded or mean ± S.D. 

### 2.5. RNA Extraction and Gene Expression Analyses

Total RNA from individuals (100 mg) at 12 and 26 dpf was extracted using TRIzol™ (Invitrogen, San Diego, CA, USA) according to manufacturer’s protocol. RNA concentration and quality were determined by spectrophotometry (NanoDrop 2000, Thermo Fisher Scientific, Madrid, Spain) measuring the absorbance at λ = 260 and 280 nm and by denaturing electrophoresis in TAE agarose gel (1.5 %), respectively. Total RNA was treated with DNase I Amplification Grade (Invitrogen, San Diego, CA, USA) according to manufacturer’s protocol and then reverse transcribed in 10 μL reaction volume containing 3 μg total RNA using the SuperScript™ First-Strand Synthesis System for RT-PCR (Invitrogen, San Diego, CA, USA) with oligo (dT) (12-18) (0.5 μg/ul) and random hexamers primers (50 ng μL^−1^), 10X RT buffer (200 mM Tris-HCl (pH 8.4), 500 mM KCL), 25 mM MgCl_2_, 0.1 M DTT, 10 mM dNTP mix, SuperScript™ II RT (50 U μL^−1^), RNaseOUT™ (40 U μL^−1^), followed by RNase H (2 U μL^−1^) (Invitrogen, San Diego, CA, USA) treatment. Reverse transcription reactions were carried out in a thermocycler (Mastercycle R nexus GSX1, Eppendorf AG, Hamburg, Germany) and run according to manufacturer’s protocol. The samples were diluted 1:20 in molecular biology grade water and stored at −20 °C until further analyses. The expression of *amy* (AC MT006358), *chy* (AC MT006344), *try* (AC MT006359), *lpl* (AC MT006346), *phl* (AC MT006345), and *pep* (AC MT006343) was analyzed in individuals at 12 and 26 dpf fed the four dietary treatments (30:15, 30:10, 45:15, and 45:10). Quantitative PCR analyses were carried out in triplicate in a 7300 Real-Time PCR System (Applied Biosystems, Roche, Barcelona, Spain). The amplification mix contained 1 μL cDNA, 0.5 μL primers (20 μM), and 10 μL SYBR Green Supermix (Life Technologies, Carlsbad, CA, USA) in a total volume of 20 μL. A negative control was included (no template control) for each set of reactions on each 96-well plate. The amplification conditions were as follows: 10 min at 95 °C, 40 cycles of 20 s at 95 °C, and 1 min at 65 °C, followed by 15 s at 95 °C, 1 min at 60 °C, 15 s at 95 °C, and finally, 15 s at 60 °C. A standard curve was obtained by amplification of a dilution series of cDNA for calculation of the efficiency (E) for each set of primers. Real-time PCR efficiencies were determined for each gene from the slopes obtained with Applied Biosystems software, applying the equation E = 10[−1/slope], where E is PCR efficiency. The relative gene expression ratio (R) for each gene was calculated according to Pfaffl’s [33] formula:

R = (E_target gene_)^ΔCq target gene (mean sample − mean reference sample)^/(E_reference gene_)^ΔCq reference gene (mean sample − mean reference sample)^,

where ΔCq is the deviation of the target sample minus the reference sample. The relative expression of the genes was normalized using glyceraldehyde-3-phosphate dehydrogenase (*GAPDH*, AC MT006341) as the reference gene since it did not exhibit any significant variation in expression between samples.

### 2.6. Digestive Enzyme Activity Assays

The activity of intestinal (alkaline phosphatase and leucine alanine peptidase), pancreatic (α-amylase, bile salt-activated lipase, trypsin, and chymotrypsin), and gastric (pepsin) enzymes was used to evaluate the impact of the four dietary treatments on the digestive system of *P. punctifer* larvae and early juveniles. Fish pancreatic and intestinal segments were dissected according to Cahu and Zambonino Infante [34] and homogenized in 30 volumes (*v/w*) of Tris-mannitol buffer (50 mM mannitol, 2 mM Tris-HCl; pH 7.5) for 30 s (Ultra-Turrax T25, Germany). Then, 100 μL of 0.1 M CaCl_2_ was added to the homogenate and the extract was sonicated (Vibra-cell^©^, Sonics, Newtown, CT, USA) for 1 min. During the homogenizing process, samples were kept on ice (0–4 °C) to reduce the enzymatic activity. An aliquot of homogenate was stored at −80 °C until analysis. The remaining homogenate was processed for intestinal brush border (BB) purification according to the recommendations of Gisbert et al. [35] in order to properly determine alkaline phosphatase activity. To do so, the homogenate was centrifuged at 9000× *g* and 4 °C for 10 min, the precipitate discarded and the supernatant centrifuged again at 34,000× *g* and 4 °C for 30 min. The pellet containing the BB of enterocytes was resuspended in 1 mL of buffer (0.1 M KCl, 5 mM Tris-HEPES, 1 mM DTT; pH 7.5) and stored at −80 °C until analysis [36]. 

Enzyme activity was measured at 12, 20, and 26 dpf according to Castro-Ruiz et al. [24]. In brief, alkaline phosphatase (AP, E.C. 3.1.3.1) activity was quantified using PNPP (4-nitrophenyl phosphate) as substrate in 30 mM Na_2_CO_3_ buffer (pH 9.8); one unit of enzyme activity (U) was defined as 1 μg nitrophenol released per minute and milliliter of BB homogenate at 27 °C and measured at λ = 407 nm [37]. Leucine–alanine peptidase (LAP, E.C. 3.4.11) was performed using leucine–alanine as substrate in 50 mM Tris-HCl buffer (pH 8.0); one unit of enzyme activity (U) was defined as 1 nmol of the hydrolyzed substrate per minute and milliliter of homogenate at 27 °C and measured at λ = 530 nm [38]. Regarding pancreatic enzymes, α-amylase (E.C. 3.2.1.1) activity was measured at λ = 580 nm using soluble starch (0.3%) dissolved in Na_2_HPO_4_ buffer (pH 7.4) as substrate [39] and its activity was defined as the milligram of starch hydrolyzed during 30 min per milliliter of homogenate at 27 °C. Bile salt-activated lipase (E.C. 3.1.1) activity was measured using 4 p-nitrophenyl myristate as substrate dissolved in 0.25 mM Tris-HCl (pH 9.0), 0.25 mM 2-methoxyethanol, and 5 mM sodium cholate buffer. The reaction was stopped with a mixture of acetone:n-heptane (5:2), the extract was centrifuged (6080× *g*, 2 min at 4 °C), and the absorbance of the supernatant was read at room temperature at λ = 405 nm. Lipase activity was defined as the micromoles of substrate hydrolyzed per minute and milliliter of homogenate [40]. Trypsin (E.C. 3.4.21.4) activity was assayed at 27 °C and measured at λ = 407 nm using BAPNA (N-α-benzoyl-DL-arginine p-nitroanilide) as substrate [41]. One U of trypsin per milliliter was defined as 1 μmol BAPNA hydrolyzed per minute and milliliter of enzyme. Chymotrypsin (EC. 3.4.21.1) activity was determined at 27 °C and measured at 256 nm using BTEE (N-benzoyl-L-tyrosine ethyl ester) as substrate in 80 mM Tris-HCl, 100 mM CaCl_2_ buffer (pH 7.2). Chymotrypsin activity corresponded to 1 μmol BTEE hydrolyzed per minute and milliliter of homogenate [42]. Pepsin assays were performed at 27 °C and at λ = 280 nm using hemoglobin as substrate [42]. Pepsin activity was defined as 1 μmol of hemoglobin liberated per minute and milliliter of homogenate after 10 min of incubation. All spectrophotometric analyses were performed as recommended by Solovyev and Gisbert [43] in order to prevent sample deterioration. Enzymatic activities were read using a microplate scanning spectrophotometer (Synergy HT, Bio-Tek, Bad Friedrichshall, Germany) and expressed as specific (mU mg^−1^ protein) and total (mU larva^−1^) enzyme activities. Soluble protein in enzyme extracts was quantified using the Bradford technique [44] using bovine serum albumin as a standard. All the assays were made in triplicate (methodological replicates).

### 2.7. Statistics

Results were expressed as mean ± S.D. (*n* = 3). All data were checked for normality (Kolmogorov–Smirnov test) and homogeneity of variance (Bartlett’s test). One-way ANOVA was performed to analyze differences in growth, proximate and fatty acid composition, histological measurements, gene expression, and enzyme activity during development and/or between dietary treatments. All pairwise multiple comparisons were performed using the Holm-Sidak method if significant differences were found at *p* < 0.05 to discriminate the significant differences. Statistical analyses were conducted using SigmaStat 3.0 (Systat Software Inc., Richmond, VA, USA).

## 3. Results

### 3.1. Growth and Survival

Although results of growth performance and survival were reported in Darias et al. [25], summary information is provided in Table 2. *P. punctifer* growth performance (TL and WW) and survival during the *Artemia* feeding period (4-12 dpf) did no show significant differences between experimental groups. However, differences in both WW and TL were observed among experimental groups at 20 and 26 dpf when fed the compound experimental diets; the groups fed the 45:15 and 30:10 diets showing the highest growth performance and the lowest WW values, respectively (*p* < 0.05). A similar trend was observed in terms of survival at the end of the experiment (26 dpf), with the groups fed the 45:15 and 30:10 P:L level diets presenting the highest and lowest values, respectively (Table 2, *p* < 0.05 [25]).

### 3.2. Proximate Composition and Fatty Acid of Diets and P. punctifer Early Juveniles

Proximate composition of diets and *P. punctifer* specimens are shown in Table 3 and Table 4, respectively. The E:P ratios were similar between the diets with the same protein content (*p* > 0.05), whereas the lower protein diets presented a higher ratio (ca. 11 kcal g^−1^ protein) than that of the higher protein diets (ca. 7 kcal g^−1^ protein) (*p* < 0.05). At 26 dpf, feeding *P. punctifer* with these diets resulted in a similar proximate composition for individuals fed both the 30:15 and 30:10 diets (53% proteins, 13% lipids, and 6% carbohydrates) (*p* > 0.05), whereas individuals fed the 45:10 and 45:15 diets displayed different protein (57% vs. 47%, respectively), lipid (11% vs. 9%, respectively), and carbohydrate (3% vs. 2%, respectively) contents (*p* < 0.05). 

Fatty acid composition of diets and *P. punctifer* specimens are shown in Table 5 and Table 6, respectively. Total lipids and total fatty acids were highest in 30:15 and 45:15 diets and lowest in the 30:10 diet (*p* < 0.05). Regarding total n-6 PUFAs, the 30:15 diet contained the highest amount (34%), followed by the 45:15 diet (27%), whereas both the 30:10 and 45:10 diets contained the lowest amounts (ca. 3%) (*p* < 0.05). The 18:2n-6 (linoleic acid, LA) fatty acid was the one accounting for these differences among diets. Total n-3 PUFAs was highest in 30:10 and 45:10 diets (42%), followed by the 45:15 (23%) and the 30:15 diets (18%) (*p* < 0.05). The 18:3n-3 (linolenic acid, LLA), 20:5n-3 (eicosapentaenoic acid, EPA), and 22:6n-3 (docosahexaenoic acid, DHA) contents in the diets accounted for the abovementioned differences. The 18:3n-3 content was highest in the 30:15 and 45:15 diets (ca. 2.4%) and lowest in the 30:10 and 45:10 diets (ca. 0.8%) (*p* < 0.05). The EPA content was highest in the 30:10 and 45:10 diets (ca. 16.5%), followed by the 45:15 diet (9%) and the 30:15 diet (6%). The highest levels of DHA were found in the 30:10 and 45:10 diets (ca. 23%) and the lowest in the 30:15 and 45:15 diets (ca. 9%). Total PUFAs was highest in both 30:15 and 45:15 diets (ca. 50%) and lowest in both 30:10 and 45:10 diets (ca. 45%) (*p* < 0.05). The ratio n-3/n-6 PUFAs was highest in the 45:10 diet (14), followed by the 30:10 diet (11) and the 30:15 and 45:15 diets (ca. 0.7) (*p* < 0.05). The ratio DHA/EPA was highest in the 30:10 diet (1.5) and lowest in the 45:15 diet (1.1) (*p* < 0.05). Both ARA/DHA and ARA/EPA ratios were similar in the four experimental diets. The ratio LA/PUFA was highest in the 30:15 diet (0.7), followed by the 45:15 (0.5) and the 30:10 and 45:10 diets (ca. 0.05) (*p* < 0.05). 

Concerning the total lipids and total fatty acid composition of *P. punctifer* specimens at 26 dpf, total lipids were similar in 30:15, 30:10, and 45:10 groups (ca. 122 mg g^−1^ DW), whereas the 45:15 group displayed a lower value (91 mg g^−1^ DW). Diets differing in the protein and lipid ratios significantly affected the fatty acid profile of fish (*p* < 0.05). Regarding total fatty acid content, individuals fed 30:15 and 30:10 diets showed the highest amount (ca. 85 mg g^−1^ DW), while those fed the 45:15 diet showed the lowest (59 mg g^−1^ DW) (*p* < 0.05). Total n-6 PUFAs was highest in fish from the 45:15 group (14%), followed by the 30:15 group (11.5%), whereas LA levels were lowest in the 30:10 and 45:10 groups (ca. 5%) (*p* < 0.05). In particular, LA content accounted for such differences, being highest in specimens from the 45:15 group (13%), followed by the 30:15 group (10%), and was lowest in the 30:10 and 45:10 groups (3%). Total n-3 PUFAs levels were highest in both 30:10 and 45:10 groups (ca. 36%), followed by the 30:15 and 45:15 groups (ca. 31%) (*p* < 0.05). Several n-3 PUFAs accounted for these differences. In particular, the LLA content was highest in the 30:15 and 45:15 groups (ca. 1.1%) and lowest in the 30:10 and 45:10 groups (ca. 0.7%) (*p* < 0.05). The EPA content was highest in the 45:10 group (10%) and lowest in the 30:15 and 45:15 groups (ca. 8%) (*p* < 0.05). The 22:5n-3 (docosapentaenoic acid, DPA) content was highest in both the 30:10 and 45:10 groups (2.3%) and lowest in the 45:15 group (1.9%) (*p* < 0.05). The DHA content was highest on the 45:10 group (22%) and lowest in the 30:15 and 45:15 groups (ca. 18%) (*p* < 0.05). 

Total PUFA content in *P. punctifer* was highest in the 45:15 group (44%) and lowest in the 30:10 and 45:10 groups (ca. 41%) (*p* < 0.05). The ratio n-3/n-6 PUFA was highest in the 30:10 and 45:10 groups (ca. 8), followed by the 45:15 (1.3) and the 30:15 (0.6) groups (*p* < 0.05). The ratio DHA/EPA was highest in the 30:15, 30:10, and 45:15 groups (2.5) and lowest in the 45:10 group (2.1) (*p* < 0.05). The ratio ARA/DHA was highest in the 30:15 group (0.1) and the rest of the dietary groups showed lower values (ca. 0.07) (*p* < 0.05). The ARA/EPA ratio was highest in the 30:15 group (0.25) and lowest in the 30:10 and 45:10 groups (ca. 0.15) (*p* < 0.05). The ratio LA/PUFA was highest in the 30:15 group (0.7), followed by the 45:15, 30:10, and 45:10 groups (0.4, 0.1, and 0.07, respectively).

### 3.3. Histological Analyses

**Figure 1:**
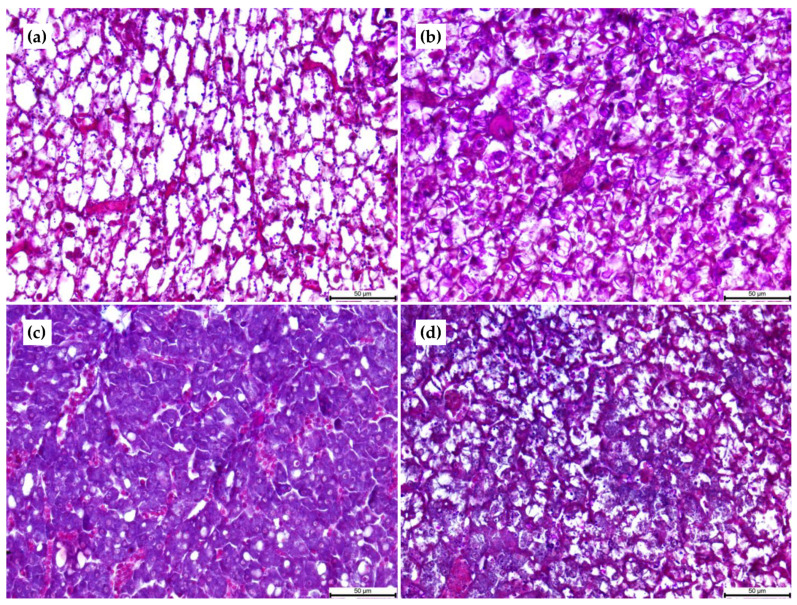
Longitudinal paraffin sections of the liver of *P. punctifer* (26 dpf) showing different levels of lipid accumulation. (**a**) Individuals fed the 30:15 diet, (**b**) individuals fed the 30:10 diet, (**c**) individuals fed the 45:15 diet, and (**d**) individuals fed the 45:10 diet. Magenta staining in the cytoplasm of hepatocytes indicates the presence of glycogen within cells. Staining: Periodic Acid Schiff (PAS)-Alcian Blue (AB) pH 2.5.

The level of lipid accumulation within hepatocytes was affected by the experimental diets (Table 7 and Figure 1).

The surface size of the lipids vacuoles was highest in the 30:15 and 30:10 groups (ca. 130 µm^2^) and lowest in the 45:15 group (20 µm^2^) (*p* < 0.05). The PAS staining revealed a differential glycogen accumulation in the liver, which was associated with the different carbohydrate content of the diets. The number of enterocytes, intestinal folds, lipid inclusion, and goblet cells; the height of enterocytes; the length of intestinal folds; and the area of the lipid droplets in the intestine are shown in Table 8.

The number of enterocytes in the AI and MI was highest in the 30:15 and 45:15 groups and lowest in the 30:10 and 45:10 groups (*p* < 0.05). However, in the PI, the number of enterocytes was highest in the 45:15 group (*p* < 0.05) and similar in the rest of the dietary groups (*p* > 0.05). A decreasing number of enterocytes was observed from the AI to the PI in the 30:15, 30:10, and 45:15 groups (*p* < 0.05), whereas the number of enterocytes was highest and similar in the AI and MI (*p* < 0.05) and lowest in the PI in the group 45:10 (*p* < 0.05). The height of enterocytes of the AI was highest in the 45:10 group and lowest in the rest of the groups, whereas the opposite was found in the MI (*p* < 0.05). In the PI, enterocytes were taller in the 45:15 group followed by the 30:15 and 45:10 groups, with the 30:10 group displaying the shortest enterocytes. In the 30:15 group, the enterocytes of the PI were taller than those from the AI, with the height of the enterocytes of the MI being in between both sizes. In the 30:10 group, enterocytes were taller in the MI and PI than in the AI (*p* < 0.05). In the 45:15 group, the height of the enterocytes decreased from the PI to the AI. In the AI, no intestinal folds were observed within the surfaces analyzed in the 30:10 group, whereas it presented the highest number of intestinal folds in the MI (*p* < 0.05), followed by the rest of the dietary groups (*p* > 0.05). No differences in the number of intestinal folds were found in the PI between groups (*p* > 0.05). The number of intestinal folds in the groups 30:15 and 45:10 was higher in the MI than in the AI and PI (*p* < 0.05). No differences in the number of intestinal folds were found between the different intestinal regions in both the 30:10 and 45:15 groups (*p* > 0.05). The length of the intestinal folds was similar in all dietary groups (ca. 195 µm) (*p* > 0.05). In the MI, folds were longest in the 30:15 group and shortest in the 30:10 group (*p* < 0.05). However, in the PI, the 45:15 and 45:10 groups presented the longest folds and the 30:10 group presented the shortest folds (*p* < 0.05). The 30:15 and 45:10 groups presented similar intestinal fold sizes between the intestinal regions. However, the 30:10 group displayed longer intestinal folds in the PI than in the other two regions, whereas, in the 45:15 group, both the AI and PI had longer folds than the MI (*p* < 0.05). Lipid deposits were only found in the PI in all dietary groups. The 45:10 and 45:15 groups presented a similar number that was higher than that of the 30:10 and 30:15 groups (*p* < 0.05). However, the size of these lipid deposits within enterocytes was similar in all dietary groups (*p* > 0.05). The number of goblet cells in the AI was highest in the 45:15 group (*p* < 0.05), with the rest of the dietary groups presenting similar values (*p* > 0.05). The number of goblet cells in the MI and PI was similar between the different dietary groups. The 30:15 group displayed a similar amount of goblet cells along the intestinal regions (*p* > 0.05), whereas the 30:10 group showed similar amount of goblet cells in the AI and MI (*p* > 0.05) that was higher than that of the PI (*p* < 0.05); the 45:15 group displayed higher number of goblet cells in the AI (*p* > 0.05) and similar amount in the MI and PI (*p* > 0.05); and the 45:10 group presented a higher number of goblet cells in the MI (*p* < 0.05) and similar amounts in the AI and PI (*p* > 0.05). 

### 3.4. Gene Expression Analyses

**Figure 2:**
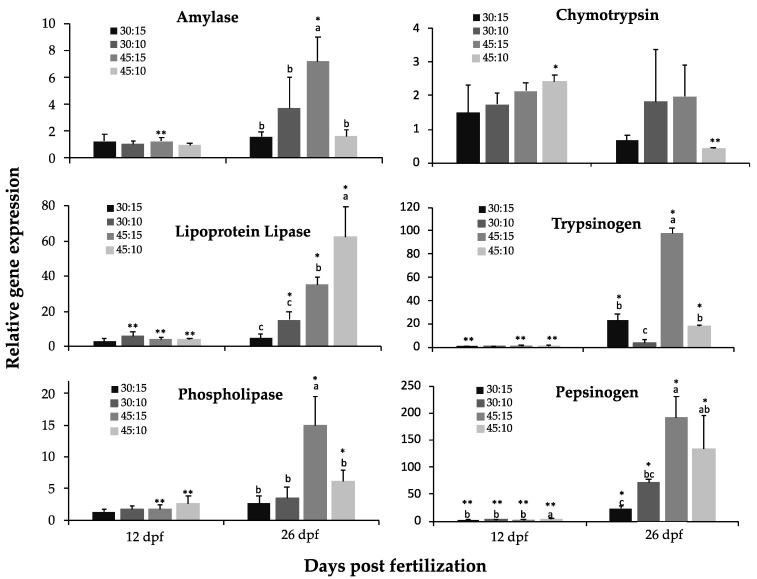
Relative expression of amylase, lipoprotein lipase, phospholipase, chymotrypsin, trypsinogen, and pepsinogen genes during the development of *P. punctifer* fed the different experimental diets containing different protein:lipid levels (in % of dry matter). Data are represented as means ± S.D. (*n* = 3). Values with a different superscript letter denote significant differences between dietary groups of the same age, and values with a different number of asterisks indicate significant differences between days for the same dietary treatment (one-way ANOVA, *p* < 0.05).

The relative gene expression of *amy*, *lpl*, *phl*, *chy*, *try,* and *pep* at 12 and 26 dpf is shown in Figure 2. During the *Artemia* feeding phase (4 to 12 dpf), no significant differences were found in the expression of the analyzed digestive genes (*p* > 0.05). 

However, digestive genes were differentially modulated by the different compound diets at 26 dpf. The expressions of *try*, *amy*, *phl,* and *pep* were highest in the 45:15 group, whereas *lpl* expression was highest in the 45:10 group (*p* < 0.05). Similar levels of *amy* and *phl* expression were observed in groups 30:15, 30:10, and 45:10 (*p* > 0.05), whereas *lpl, try, and pep* expression were lowest in the 30:15 and 30:10 groups (*p* < 0.05). The lowest expression for *try* was found in the 30:10 group followed by the 30:15 and 45:10 groups, whereas in the case of *pep*, the lowest expression was found in the 30:15 and 30:10 groups followed by the 45:10 group (*p* < 0.05). No significant differences were found in *chy* expression among the four dietary treatments (*p* > 0.05). *Try* and *lpl* expressions increased during development in all groups except in the 30:10 and 30:15 groups, respectively (*p* < 0.05). *Phl* expression increased during development in the 45:15 and 45:10 groups, whereas it remained stable in the others. *Chy* expression decreased during development in the 45:10 group, and *amy* expression increased during development in 45:15 group, whereas their expressions remained invariable in the rest of the groups (*p* < 0.05). *Pep* expression increased during development in all dietary groups (Figure 2; *p* < 0.05).

### 3.5. Digestive Enzyme Activity Analyses

**Figure 3:**
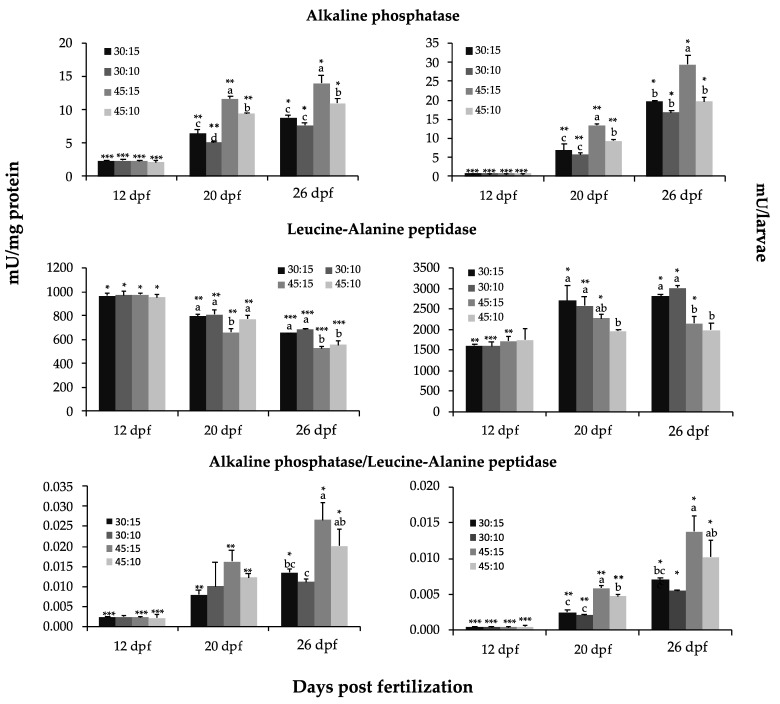
Specific (mU mg^−1^ protein, on the left) and total (mU larva^−1^, on the right) activity of brush border (alkaline phosphatase) and cytosolic (leucine–alanine peptidase) intestinal enzymes and the ratio alkaline phosphatase to leucine–alanine peptidase ratio in *P. punctifer* (12, 20, and 26 dpf) fed the different experimental diets containing different protein:lipid levels (in % of dry matter). Data are represented as means ± S.D. (*n* = 3). Values with a different superscript letter denote significant differences between dietary groups of the same age, and values with a different number of asterisks indicate significant differences between days for the same dietary treatment (one-way ANOVA, *p* < 0.05).

The specific and total activities of the analyzed brush border (AP) and cytosolic (LAP) intestinal enzymes are shown in Figure 3. The specific and total AP activities were similar in all dietary treatments at 12 dpf (*p* > 0.05), whereas they were higher in the 45:15 group at 20 and 26 dpf (*p* < 0.05). The specific and total AP activities significantly increased during development in all dietary groups (*p* < 0.05). The specific and total LAP activities were similar in all dietary groups at 12 dpf (*p* > 0.05) and lowest in the 45:15 and 45:10 groups at 26 dpf (*p* < 0.05). The specific LAP activity decreased during development in all dietary groups (*p* < 0.05). In the 30:15 and 45:15 groups, total LAP activity increased from 12 to 20 dpf and then remained constant. In the 30:10 group, total LAP activity increased during the experimental period, and it remained invariable during development in the 45:10 group (*p* > 0.05). The high protein groups displayed higher specific AP/LAP ratio at 26 dpf and higher total AP/LAP ratio at both 20 and 26 dpf than the low protein groups (*p* < 0.05).

**Figure 4:**
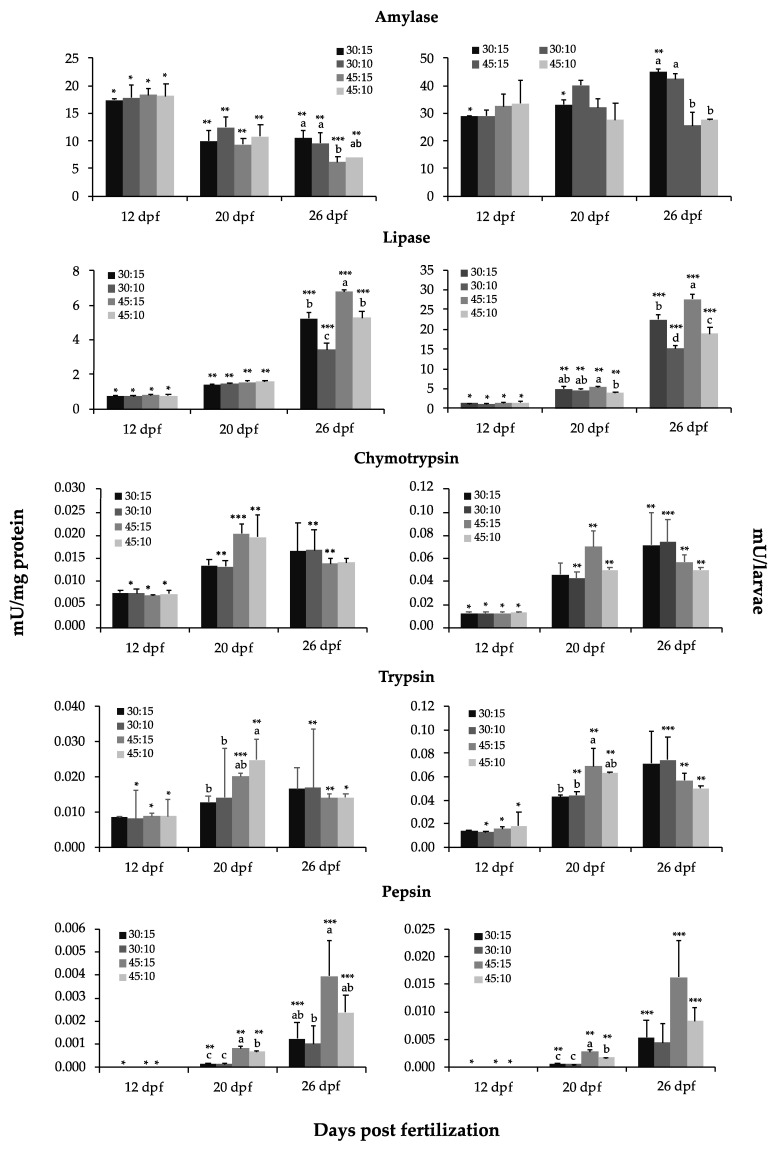
Specific (mU mg^−1^ protein, on the left) and total (mU larva^−1^, on the right) activity of α-amylase, bile salt-activated lipase, chymotrypsin, trypsin, and pepsin in *P. punctifer* (12, 20, and 26 dpf) fed the different experimental diets containing different protein:lipid levels (in % of dry matter). Data are represented as means ± S.D. (*n* = 3). Values with a different superscript letter denote significant differences between dietary groups of the same age, and values with a different number of asterisks indicate significant differences between days for the same dietary treatment (one-way ANOVA, *p* < 0.05).

The specific and total activities of α-amylase, bile salt-activated lipase, chymotrypsin, trypsin, and pepsin are shown in Figure 4. The specific and total activities of α-amylase were similar in all dietary groups at 12 and 20 dpf (*p* > 0.05). However, at 26 dpf, specific and total α-amylase activities were highest in the 30:15 and 30:10 groups, and lowest in the 45:15 group (*p* < 0.05). Specific α-amylase activity decreased from 12 to 20 dpf in all dietary treatments to remain constant afterwards, with the exception of the 45:15 group, whose specific α-amylase activity continued to decrease at 26 dpf (*p* < 0.05). Total α-amylase activity remained constant during the experimental period with the exception of the 30:15 group, which showed an increase in activity from 20 to 26 dpf.

The specific and total activities of bile salt-activated lipase were similar in all dietary groups at 12 dpf (*p* > 0.05). At 20 dpf, specific activity continued to be similar in all dietary groups, whereas total activity was highest in the 45:15 group (*p* < 0.05). At 26 dpf, specific and total bile salt-activated lipase activities were highest in the 45:15 group and lowest in the 30:10 group (*p* < 0.05). Both specific and total bile salt-activated lipase activities increased during development in all dietary groups (*p* < 0.05).

The specific and total activities of chymotrypsin were similar in all dietary groups throughout the experimental period (*p* > 0.05). Chymotrypsin-specific activity remained invariable during development in the 30:15 group (*p* > 0.05), and total activity increased during development in the 30:10 group (*p* < 0.05). In the rest of dietary groups, chymotrypsin-specific and total activities increased from 12 to 20 dpf and remained constant thereafter (*p* > 0.05).

The specific and total activities of trypsin were similar in all dietary groups at 12 dpf (*p* > 0.05). At 20 dpf, specific and total trypsin activities were highest in the 45:10 and 45:15 groups, respectively, and lowest in both the 30:15 and 30:10 groups (*p* < 0.05). At 26 dpf, the level of specific and total trypsin activities was similar in all dietary groups (*p* > 0.05). The level of specific trypsin activity was constant during development in the 30:15 group (*p* > 0.05). In the 30:10 group, an increase in specific trypsin activity was observed between 12 and 26 dpf (*p* < 0.05). In the 45:15 and 45:10 groups, specific trypsin activity increased from 12 to 20 dpf and decreased afterwards (*p* < 0.05). Total trypsin activity increased during development in the 30:10 group, whereas it increased from 12 to 20 dpf in both 45:15 and 45:10 groups to remain constant afterwards.

The specific and total activities of pepsin were similar in all dietary groups at 12 dpf (*p* > 0.05). At 20 dpf, specific and total pepsin activities were highest in the 45:15 group, and lowest in the 30:15 and 30:10 groups (*p* < 0.05). At 26 dpf, specific pepsin activity continued to be highest in the 45:15 group and lowest in the 30:10 group (*p* < 0.05), whereas total activity was similar in all dietary groups (*p* > 0.05). The specific and total pepsin activities were constant during development in the 30:10 group (*p* > 0.05) and increased during development in the rest of dietary groups (*p* < 0.05).

## 4. Discussion

The feeding protocol used in this study was adapted to the digestive capacities of developing *P. punctifer*. Thus, individuals were weaned at 13 dpf from live prey (*Artemia* nauplii) to the different experimental compound diets when the morphological and functional development of the digestive system was achieved [23,24], moment that we consider, from a digestive physiology point of view, the beginning of the juvenile stage in this species. 

### 4.1. The Influence of Dietary Protein:Lipid:Carbohydrate Content and Ratios in P. punctifer Performance

This study aimed at understanding the physiological mechanisms underlying the different developmental responses observed in fish fed diets differing in their protein, lipid, and carbohydrate levels and ratios [25]. Four experimental diets were formulated with two different protein (30% vs. 45%) and lipid (10% vs. 15%) contents. Consequently, carbohydrates were used to complete the formula, with the low-protein diets having higher carbohydrate amounts (>25%) than the high-protein diets (<8%). The differences in growth and survival among experimental groups were primarily associated to the dietary protein content and, secondly, to the lipid level. The poorer growth performance in 30:10 and 30:15 groups indicated insufficient protein availability for maintaining proper growth and metabolism in *P. punctifer* early juveniles and insufficient and/or inadequate energy sources (lipids and carbohydrates) to compensate this protein deficiency.

As the carbohydrate content in the low-protein diets was significantly higher, their influence on the overall physiology of fish fed these diets cannot be neglected. In fact, both glycogen and lipid deposits in livers were correlated with the carbohydrate content of the diets. High carbohydrate levels (>30%) are known to increase lipid deposition in the liver of fish by enhancing lipogenesis and lipid uptake potential [12,17,45]. Moreover, fish feeds with an excess of carbohydrate content may lead to unbalanced fat depositions, suppressed immune function, and reduced health [10]. Excess carbohydrates are converted into simple sugars by digestion and excess glucose may be stored as glycogen (glycogenesis) or converted into lipids (lipogenesis) [46,47,48,49], playing a key role in nutrient retention. The increased glycogen and lipid deposits observed in the livers of individuals fed the 30:10 and 30:15 diets suggest that both glycogenesis and lipogenesis could have been occurred in the liver to regulate glucose homeostasis in *P. punctifer*. Further research on the regulation of glucose metabolism-related genes is needed to elucidate the carbohydrate metabolism in *P. punctifer* and to better determine the optimal dietary carbohydrate:lipid (C:L) ratio favoring protein sparing and overall physiological performance in this species. Indeed, although all diets were isoenergetic, high C:L ratio reduced growth, indicating that different energy sources influence the utilization of lipids and carbohydrates. This is because of the interconnection between these energy sources through the potential lipid conversion into glucose via gluconeogenesis and the potential glucose conversion into lipids via lipogenesis. Similar results have also been observed in other fish species [6,10,50]. Digestion and metabolism of dietary carbohydrates are closely associated to the feeding habits of the fish, with omnivorous and herbivorous fish being able to better utilize dietary carbohydrates [51,52,53] than carnivorous fish. This study showed that *P. punctifer* has a clear preference for lipids as a source of energy rather than carbohydrates. As observed in rainbow trout [17], dietary lipids had a better protein-sparing effect than carbohydrates at a similar level of digestible energy intake in *P. punctifer,* as shown by the better growth and survival of individuals fed the 45:15 diet (that contained 2% carbohydrates) compared to the individuals fed the 45:10 diet (that contained 8% of carbohydrates). On the contrary, a preference of carbohydrates over lipids as a source of energy has been observed in some omnivorous freshwater fish [6,54]. Based on the overall results, the optimal dietary C:L ratio for *P. punctifer* was between 0.2 (45:15 diet) and 0.8 (45:10 diet).

The proximate composition of the whole body of *P. punctifer* was modulated by the different dietary protein:lipid:carbohydrate levels. The whole-body protein was the same in all dietary groups, which means that individuals fed the low-protein diets had to synthesize more proteins than those that had protein provided through the diet to enable them to reach the required amounts in their tissues (ca. 45%). On the contrary, the body protein content has been found to be positively correlated to the dietary protein content in other fish species [55,56,57,58,59,60]. The groups fed the low-protein diets presented higher carbohydrate content than the other dietary groups, and the fact that individuals fed the 45:15 diet presented the lowest carbohydrate and lipid content suggests that the E:P and/or C:L ratio promoted higher lipid accumulation in the other dietary groups. A dietary modulation of the body composition has also been observed in juveniles of tilapia *Oreochromis niloticus* × *O. aureus* [18], whereas no effect was observed in the Nile tilapia *O. niloticus* during the grow-out phase [4]. This could indicate differences in the regulation of nutrient metabolism depending on the species and developmental stages. Indeed, it has been suggested that anabolism and catabolism processes could take place in later stages of development to stabilize the proximate composition in whole body [4].

The E:P ratio can affect growth performance, feed efficiency, and body composition of fish. In this study, the growth performance and metabolism of *P. punctifer* were significantly affected by the dietary E:P ratio of the experimental diets. In particular, an E:P ratio of 11 kcal g^−1^ protein in the 30:15 and 30:10 diets induced lipid deposition in the liver and decreased somatic growth. Higher liver (or body) lipid content with increasing E:P ratio has been also observed in other fish species such as in grass carp *Ctenopharyngodon idella* [61], Chinese perch *Siniperca chuatsi* [3], *O. aureus* [1], and channel catfish *Ictalurus punctatus* [62]. A similar optimal E:P ratio for growth and overall performance to that found for *P. punctifer* (ca. 7 kcal g^−1^ protein) has been observed in Asian seabass *Lates calcarifer* [63], African sharptooth catfish *Clarias gariepinus* [64], and grass carp [61], whereas the higher E:P ratio that was suboptimal for *P. punctifer* (11 kcal g^−1^ protein) resulted to be optimal for channel catfish [62]. In *P. punctifer,* protein levels in the high E:P diets (30:10 and 30:15) were below the optimal level for this species and stage of development and were mostly used for body protein maintenance; and dietary lipids and carbohydrates were not used efficiently or were lacking in quantity for energetic purposes. In addition, a possible dietary starch interaction with protein digestibility cannot be neglected in these high-carbohydrate dietary groups, as a decrease in protein digestibility related to an increase in dietary starch content increase has been demonstrated in other fish [65]. The high carbohydrate content of these diets might have contributed to provide energy, as shown by the increased levels of α-amylase activity in these groups, although they did not contribute with sufficient efficiency to promote growth. Reduced growth as the result of overloading of carbohydrates in the fish diet has been observed in other fish species [17,45,66]. Therefore, low protein synthesis results in low growth performance and poor metabolism, whereas the opposite trend occurs when fish are fed with low E:P ratio diets [3].

If a diet is deficient in an essential nutrient, fish consume more feed to fulfill the demands for that specific nutrient [3]. In line with this, it has been shown that fish fed high carbohydrate diets need to increase their feed intake in order to gain adequate amino acids levels for promoting growth [4]. Assuming that this was also the case of *P. punctifer* fed the 30:10 and 30:15 diets, it can be hypothesized that energy supplied in these low-protein diets was insufficient in quantity and/or quality to compensate for the extra energy costs associated to deamination as well as to swimming and foraging behaviors; consequently, reducing the energy available for growth. Among the tested diets, the lower the E:P ratio the better growth and survival in *P. punctifer*. The opposite has been observed in the omnivorous Nile tilapia [4] and also in carnivorous fish such as in the black sea bream *Sparus macrocephalus* [60]. This has been attributed to an excess of dietary protein that would result in the loss of energy for deamination of amino acids toward protein catabolism [4].

### 4.2. The Influence of Dietary Fatty Acid Composition in P. punctifer Body Fatty Acid Composition

Concerning the fatty acid composition of the different dietary groups, the DHA content of individuals from the 30:10 and 45:10 groups (ca. 23%) reflected that of the ingested diet (ca. 21%) and accounted for the majority of the total n-3 PUFA tissue content. However, fish fed the 30:15 and 45:15 diets, which contained lower DHA levels (ca. 9%), displayed considerably higher DHA content (ca. 18%). This suggests that *de novo* DHA synthesis occurred in those dietary groups, which is in accordance with the depletion of α-linolenic acid (LLA), the precursor of DHA biosynthesis, observed in these groups [67]. The DHA content in individuals from the 30:15 and 45:15 groups was significantly lower (ca. 18%) than that from the 30:10 and 45:10 groups (ca. 23%). These results suggest that the higher dietary DHA led to seemingly higher DHA levels in tissues than required.

As in the diets, LA accounted for the differences observed in the total n-6 PUFA content in fish. The total n-6 PUFA content was between 5 and 7 times higher in 30:15 and 45:15 diets compared to 30:10 and 45:10 diets. This profile in total n-6 PUFA content was also found in the dietary groups, although the differences were lower (ca. 2 times). When comparing the total n-6 PUFA content of the diet with that of the tissues of each dietary group, the total n-6 PUFA content (and so LA) remained the same in the 30:10 and 45:10 groups, whereas it was 2 and 3 times lower in the 45:15 and 30:15 groups, respectively. This indicates that higher dietary n-6 PUFA (meaning LA) content enabled the 30:15 and 45:15 groups to use some for physiological functioning and to stock a higher amount in their tissues than that allowed by the 30:10 and 45:10 diets. In addition, it appeared essential for 30:10 and 45:10 groups to retain the entirety of the low n-6 PUFA content (ca. 5%) of the 30:10 and 45:10 diets in their tissues. Since the 45:10 group displayed the second highest growth of the groups, it seems that a lower n-6 PUFA tissue content does not compromise the growth of specimens. The 30:15 and 45:15 groups displayed lower LA values than those of the diets, although it did not result in an increased ARA content. The different use of LA between both dietary groups resulted in the 45:15 group presenting the highest total n-6 PUFA content. The sum of total n-3 and n-6 PUFA contents of each dietary group led to a higher total PUFA content in 45:15 group and lower in the 30:10 and 45:10 groups, with the 30:15 group displaying intermediate values, although the differences in content were not very large (43% vs. 40%). The only clear differences between the group with the highest growth (45:15) and the rest of the dietary groups were the n-3/n-6 PUFA, the LA/PUFA, and the LLA/PUFA ratios for which the 45:15 displayed intermediate values. However, these profiles alone do not account for the differences in growth as the 45:10 group, which presented the second highest growth, displayed the highest n-3/n-6 ratio together with the lowest LA and LLA/PUFA ratios. The subtle balance between all the components in the 45:15 diet certainly accounted for the improved results observed in *P. punctifer* early juveniles in the present study, since the other diets that induced less efficient development had one or several compounds in a different proportion as compared to the composition of the 45:15 formula.

Contrary to marine fish [68], a dietary DHA:EPA ratio closer to 1 likely contributed to an improved performance in *P. punctifer*, as was the case of the 45:15 diet. Independently of the DHA:EPA ratio provided in the diets, *P. punctifer* early juveniles contained a similar DHA:EPA ratio in their tissues (ca. 2.5), suggesting that this is the proportion needed to maintain the correct physiological homeostasis. Similarly, the DHA:EPA ratio was consistently higher in the triacylglycerols deposited in farmed Atlantic salmon than in the fish oils used in salmon feeds [69]; and, in Atlantic salmon, the DHA:EPA ratio was also increased in the muscle compared to that of the diet, consistent with the selective oxidation of EPA, thereby resulting in a selective retention of DHA by the fish [67]. *P. punctifer* seemed to not depend on EPA or DHA, but rather on LLA to promote growth, as observed in the 30:15 and 45:15 groups, where the dietary LLA was used to synthesize DHA to meet the tissue requirements vital for cell membrane structure and function (ca. 18% TFA). However, when dietary DHA levels were high enough for its tissue requirements, as it was the case of the 30:10 and 45:10 diets, then, the *de novo* DHA biosynthesis pathway from its precursor LLA seemed to be inhibited. Other freshwater fish such as the African sharptooth catfish and Tilapia zilli *Coptodon zillii* also convert C18 PUFA to HUFA and, in the case of the African catfish, high or low dietary n-3 HUFA levels have been shown not to influence growth [70,71]. The latter could not be confirmed in *P. punctifer* as all experimental diets contained high DHA levels (9–24% TFA). Due to the low protein and lipid content of the 30:10 diet, the lipids were mostly used to maintain the adequate lipid and fatty acid levels in tissues, which was similar to those of the other dietary groups, rather than for promoting somatic growth.

### 4.3. The Influence of Dietary Composition in the Digestive Function of P. punctifer

The number of hepatocytes in the liver was in line with the degree of physiological development of the individuals fed the different dietary treatments. Thus, individuals fed the 45:15 diet presented the highest number of hepatocytes and those from the 30:10 group the lowest. In terms of hepatic lipid accumulation, individuals fed the low-protein diets (30:15 and 30:10) showed the fattiest livers, likely due to an excess of dietary carbohydrate promoting lipogenesis in the liver [12,45,72,73]. As mentioned before, this pattern was also reflected in the total lipid and total fatty acid content of individuals. In particular, individuals from the 30:10 dietary group, although fed the lowest lipid (and protein) content, accumulated lipids and fatty acids similar to the levels observed in the 30:15 and 45:10 groups. A similar liver metabolic response was found in Nile tilapia, where lipogenesis increased in the liver when lipid intake was limited and *de novo* fatty acid synthesis increased the hepatic fatty acid content [73]. In Nile tilapia, the stimulated glycolysis by either dietary lipid deficiency or high dietary carbohydrate was suggested to provide the substrates for lipogenesis [73]. A similar situation could be happening in *P. punctifer* fed the 30:15 and 30:10 diets. The reduction in capacities for triglyceride and phospholipid hydrolysis in these individuals, as the reduced *lpl* and *phl* expression showed, could be indicators of the altered lipid metabolism observed in these individuals, hypothesis supported by the large accumulation of lipids in hepatocytes observed histologically. In addition, a decrease in lipase activity was observed in these dietary groups, which was correlated with the lipid inclusion levels in the posterior intestine. The reduced lipase activity observed in the 30:10 group together with the reduced number and area of lipid droplets in the posterior intestine suggests an alteration of the lipid digestion and absorption at the intestinal level in these individuals. The 30:10 diet seemed to have insufficient lipid and excessive carbohydrate content for *P. punctifer* early juveniles. The increase in α-amylase activity together with a decrease in bile salt-activated lipase activity and *lpl* expression suggests that both macronutrients are inducing an altered lipid metabolism. Similar findings have been observed in rats were increased levels of insulin produced by raised levels of glucose increased pancreatic α-amylase activity together with a decrease in chymotrypsinogen and lipase activities [74]. It has been shown in humans that *lpl* expression is more stimulated by carbohydrates than by lipids [75]. The *lpl* expression was particularly low in groups fed the 30:15 and 30:10 diets, which could be related to their higher dietary carbohydrate levels but also to their low protein content. Indeed, low dietary protein content has shown to decrease LPL activity and impair VLDL-TAG export from the liver in rats [76]. In line with this, the lower level of *lpl* gene expression observed in *P. punctifer* individuals fed the 30:10 and 30:15 diets could be indicating a limited VLDL export from the liver resulting in the development of fatty livers in these specimens. Moreover, protein deficiency can induce insulin resistance associated with reduced LPL activity, overproduction of TAG, and impaired VLDL catabolism [77]. In humans, insulin downregulates *lpl* expression in insulin-resistant individuals, which present an altered intracellular lipid metabolism [78]. Diabetics display a decrease in insulin following a high-carbohydrate diet [79]. In some fish, such as in rainbow trout, insufficient insulin secretion occurred when fed a high-carbohydrate diet [48]. It is clear from this study that both carbohydrate and protein contents of the 30:10 and 30:15 diets were inadequate for *P. punctifer.* However, further research is needed to decipher the involvement and the interconnectivity of the mechanisms of action of these macronutrients in regards to the hepatic lipid metabolism in these specimens and whether these low-protein high-carbohydrate diets induce insulin resistance or not in *P. punctifer*. Concerning the 45:10 and 45:15 groups, the differences in the lipid:carbohydrate content ratio of the provided diets (1 vs. 5, respectively) could be responsible for the higher *lpl* expression in the 45:10 group compared to the 45:15 group. A higher dietary carbohydrate intake may have a stronger effect on fat storage in the liver, as observed in individuals from the 45:10 group in comparison to the 45:15 group. The higher *lpl* gene expression levels observed in the 45:10 and 45:15 groups reflect a better lipid transport from the vascular system to tissues, and the lowest lipid accumulation in the liver of these dietary groups indicates a more balanced lipid metabolism than the rest of the dietary groups. This was in line with the level of intestinal maturation of these groups compared to the others.

The activity of the pancreatic and intestinal enzymes are useful and reliable markers for assessing the development and state of the digestive function in fish [80]. As in many other fish, the specific activity of AP increases concomitantly with the decrease in the activity of LAP during the larval development of *P. punctifer* [24]. In this study, the ratio AP/LAP at 26 dpf measured in the four dietary groups indicated a lower degree of maturation of the intestine in the 30:10 and 30:15 groups, whereas the most mature intestine corresponded to individuals from the 45:15 group. These results indicate that the different dietary regimes were directly affecting the developing process of *P. punctifer*, which was also demonstrated at the histological level. Differences were particularly evident in regards to the number of enterocytes along the different parts of the intestine, the number and length of the intestinal folds, the degree of accumulation of lipids in the PI, and the number of goblet cells in the AI. In particular, the smaller the difference in the number of the enterocytes between the intestinal parts, the better the overall performance, as observed in the 45:15 group. In this sense, and considering the present results, a ratio of the number of enterocytes in the AI to the PI higher than 1.2 could be considered an indicator of a developmental delay in this species. These histological observations concerning the intestinal development were correlated with several indicators of the digestive function, such as the AP/LAP, bile salt-activated lipase and pepsin activities, and growth. In terms of lipid metabolism, the 30:15 and 30:10 groups presented the lowest number of lipid deposits in the PI, which is interpreted as an indicator of a delay in intestinal maturation in these groups [81]. In addition, both the 30:15 and 30:10 groups presented the fattiest livers of all groups, which, as mentioned before, seems to be associated to the high carbohydrate level contained in these diets. Further, the higher number of goblet cells found in the AI of the 45:15 group was correlated with the improved development and growth in these specimens, as one of the various roles of neutral mucins secreted by goblet cells is to contribute to the digestion and transformation of the food into chyme, as well as to the absorption of easily digestible molecules such as disaccharides and short-chain fatty acids [82].

The dietary proximate composition differently modulated the gene expression of pancreatic and gastric enzymes as well as the post-transcriptional regulation of the enzyme production. Thus, *amy* expression was higher in 45:15 group compared to the rest of the groups, whereas α-amylase activity displayed the opposite trend. These results suggest that α-amylase activity was indeed modulated at the post-transcriptional level in all dietary groups according to the carbohydrate content of the diets. Thus, the groups fed the 30:10 and 30:15 diets, which contained the highest carbohydrate levels (31% and 25%, respectively), showed the highest amylase activity. A similar response has been observed in European sea bass *Dicentrarchus labrax* [83]. From the data obtained on gene expression and enzyme activity regulation from the different dietary groups, it seems that *P. punctifer* juveniles have the capacity to respond to such high levels of carbohydrates by increasing α-amylase activity to a certain extent. Indeed, they accumulated higher carbohydrate content in their tissues than the other dietary groups. However, the excessive lipid accumulation in livers of these individuals suggests that the carbohydrate content was excessive in these diets. Moreover, the low level of *amy* expression observed in the 30:10 and 30:15 groups, and even in the 45:15 group, could be indicating a negative regulation of gene expression in response to excessive dietary carbohydrate levels.

The diets with the higher lipid content promoted the development of the digestive system, as reflected by the higher number of enterocytes in the AI and MI found in the 30:15 and 45:15 groups. This could explain the better growth of the 30:15 group compared to the 30:10 group, despite the same protein content. This is also supported by the higher level of bile salt-activated lipase activity of the 30:15, which was similar to that of the 45:10 group, compared to the 30:10 group. The level of this lipolytic enzyme is generally modulated by the dietary lipid content [84]. However, in the present study, the 45:15 group displayed a higher level of lipase activity than the 30:15 group, which could be explained by the fact that, although the total lipid content was similar in both diets, the 45:15 diet contained higher amount of TAG than the 30:15 diet [25], as well as to a more mature digestive system. As observed in the low-protein groups, lipase activity seems to also be modulated by the protein content in the high-protein groups, since the 45:10 group presented higher lipase activity than the 30:10 group (which also presented similar fatty acid composition) and similar activity to the 45:15 and 30:15 groups.

The efficiency of utilization of dietary PUFAs depends on the dietary lipid class (neutral lipids or phospholipids). The lipid ingredients used to formulate the four diets were soybean and marine lecithin as sources of neutral lipids and phospholipids, respectively. The early stages of fish have high requirements for phospholipids [85], which are essential nutrients for growth, survival, and maturation of the intestinal functions [85,86]. The gene expression level of *phl* was correlated with the phospholipid content of the diets. The higher phospholipid content of the 45:15 diet compared to the 45:10 diet could also account for the better growth and performance of individuals fed the 45:15 diet, as well as to changes in hepatic lipid accumulation. Furthermore, the higher phospholipid content of the 30:15 diet could contribute to the similarity in measures of TL and WW (at 20 dpf) with the 45:10 group, despite the considerably lower amount of available dietary protein. Phospholipids are especially important in marine fish, as they use the dietary n-3 PUFAs from the phospholipid fraction more efficiently than those from the neutral lipid fraction [87]. Similarly, it has been shown in some freshwater fish, such as the African sharptooth catfish and goldfish *Carassius auratus*, that phospholipids are the main lipids catabolized [70,88]. Although the lipid class preference of *P. punctifer* has not been determined, the higher phospholipid content of the 30:15 diet compared to the rest of the diets could have accounted for promoting growth in the individuals of this group to the levels of the 45:10 group, despite the reduced amount of dietary protein.

The level of *try* expression was higher in the 45:15 group at 26 dpf, whereas trypsin activity was similar in all four dietary groups at that age. However, differences in trypsin activity were observed at 20 dpf; the groups fed the higher dietary protein content showing the highest level of activity. These results indicate that while *try* and *pep* were both contributing to protein digestion at 20 dpf, and, consequently, a modulation of the enzyme activity depending on the dietary regime is observed, pepsin activity increased at 26 dpf and trypsin activity levels remained stable in time and invariable among dietary regimes. At 26 dpf, *try* expression was highest in the 45:15 group and lowest in the 30:10 group. Unexpectedly, the level of *try* expression in the 45:10 group was lower than that of the 45:15 group, but similar to that of the 30:15 group. These findings suggest that, in *P. punctifer*, the modulation of *try* expression not only depends on the dietary protein level, but rather on the combination of nutrients. Indeed, it has been shown in rats that *try* expression increases when a protein-free carbohydrate-rich diet is offered [89]. Moreover, in cod *Gadus morhua* (fed to satiation), trypsin was the only enzyme among those examined by Lemieux and colleagues [90] that could potentially limit food conversion efficiency, therefore, limiting growth rate. Overall, in cod, trypsin showed a stronger correlation with growth rate than with food intake, and in *P. punctifer,* the high protein diet gave the best results for growth and highest *try* expression at 26 dpf [90]. The levels of trypsin activity were similar in all dietary groups indicating a post-transcriptional regulation [83]. The similar level of trypsin activity among the four dietary groups indicates that similar levels are used to digest protein content ranging from 30% to 43% and/or that there are other nutrients inducing the activity of this enzyme in the low-protein groups. Further research on the influence of carbohydrates in the regulation of trypsin activity as well as on the involvement of the digestive hormone cholecystokinin in the regulation of *try* secretion [91] will contribute to better understand the regulation of trypsin activity in this species.

*Pep* expression was found to be modulated by the dietary protein content. Thus, high-protein dietary groups displayed higher levels of *pep* expression than the low-protein dietary groups. At the enzyme level, however, the 45:15 group showed the highest pepsin activity and the 30:10 group the lowest, with the 45:10 and 30:15 group displaying intermediate values. These results could be related to the high interindividual variability obtained in the enzymatic assays that resulted in a nonsignificant differentiation of pepsin activity between those dietary groups, although it was noted that pepsin activity tended to be higher in the 45:10 group than in the 30:15. Alternatively, these results could show a post-transcriptional regulation of this gene to adapt to a specific nutrient combination, although some authors support that pepsin activity is poorly modulated by dietary protein [92]. In this hypothetical case, in addition to protein content, the different energy content of the 45:10 and 30:15 diets could be accounting for a similar level of pepsin activity. However, further knowledge on the nutrient’s interaction and communication with the different enzymatic machinery is needed to confirm this and to better understand the complex mechanisms underlying nutrition in fish.

## 5. Conclusions

This study showed that protein followed by lipid content are the main drivers for successful growth and survival in *P. punctifer* early juveniles. The dietary protein:lipid:carbohydrate levels and ratios influenced the whole body proximate composition, the digestive physiology and development, and hence growth, as well as survival. Individuals fed the 45:15 diet grew and survived best. This diet promoted the most rapid development of the digestive system as demonstrated by histological and functional indicators: higher number of hepatocytes, higher number of goblet cells in the anterior intestine, higher number of enterocytes in all intestinal parts, and longer folds in the posterior intestine; highest *amy*, *lpl*, *phl*, *try,* and *pep* expression; and highest lipase and pepsin activity and higher AP:LAP ratio. Altogether, this enabled individuals to better digest, absorb and metabolize nutrients. In addition to the protein content, phospholipids seemed to be key for the improved performance of the 45:15 group. It appears that a better use of the phospholipid fraction enabled this group to take advantage of all the benefits that these lipids have for growth, development, and behavior [25].

This study also found that growth performance and metabolism of *P. punctifer* were significantly affected by the dietary E:P ratio. At high E:P ratios (e.g., 11), energy was used to assure body protein requirements and the remaining energy did not contribute efficiently enough to promote growth. In addition, results indicated that *P. punctifer* has a clear preference for lipids as source of energy rather than carbohydrates, with lipids even promoting a protein-sparing effect at adequate E:P ratio. Moreover, dietary carbohydrate content higher than 25% appeared to be excessive for this species, leading to unbalanced lipid metabolism and fat deposition in livers. According to the present study, a 0.2–0.8 C:L ratio is optimal for *P. punctifer*. In view of the current findings and of the large gap in carbohydrate content between the best and the worst tested diets (2% vs. 31%) in terms of growth and survival, further research on the regulation of glucose metabolism-related genes is needed to elucidate the carbohydrate metabolism in *P. punctifer* and to better determine the optimal dietary C:L ratio that favors protein sparing and overall physiological performance in this species.

## Figures and Tables

**Table 1:** Composition of the experimental diets. Dietary treatment codes correspond to the protein:lipid level included in tested diets. DM, dry matter.

Ingredients ^1^ (in % DM)	Dietary Treatments			
	30:15	30:10	45:15	45:10
Fishmeal	36	36	53	53
Hydrolyzed fishmeal (CPSP)	9	9	14	14
Lipids	14	8	12	7
Marine lecithin	3	8	3	7
Soybean lecithin	11	0	9	0
Gelatin	15	15	15	15
Wheat starch	20	26	0	5
Vitamin mix ^2^ (x4)	2	2	2	2
Mineral mix ^3^	3	3	3	3
Betain	1	1	1	1

^1^ All dietary ingredients obtained commercially. Fishmeal hydrolysate CPSP 90:10% lipids; soluble fish protein concentrate (Sopropêche, Boulogne sur Mer, France); soy lecithin (Ets Louis François, St Maur des Fosses, France); marine lecithin LC 60 (Phosphotech, St Herblain, France). ^2^ Composition per kilogram of vitamin mixture: choline chloride 60%, 333 g; vitamin A acetate, (4000 IU g^−1^) 2 g; vitamin D3 (1920 IU g^−1^) 0.96 g; vitamin E (40 IU g^−1^) 20 g; vitamin B3 2 g, vitamin B5 4 g; vitamin B1 200 mg; vitamin B2 80%, 1 g; vitamin B6 600 mg; vitamin B9 80%, 250 mg; vitamin concentrate B12 (10 g kg^−1^), 0.2 g; biotin, 1.5 g; vitamin K3. 51%, 3.92 g; meso-inositol 60 g; cellulose, 543.3 g. ^3^ Composition per kilogram of mineral mixture: 90 g KCl, 40 mg KIO_3_, 500 g CaHPO_4_ 2H_2_O, 40 g NaCl, 3 g CuSO_4_ 5H_2_O, 4 g ZnSO_4_ 7H_2_O, 20 mg CoSO_4_ 7H_2_O, 20 g FeSO_4_ 7H_2_O, 3 g MnSO_4_ H_2_O, 215 g CaCO_3_, 124 g MgSO_4_ 7H_2_O, and 1 g NaF.

**Table 2:** Growth in terms of total length and wet weight at 12, 20, and 26 dpf, and survival at the end of the *Artemia* and compound diet feeding periods (12 and 26 dpf) of *P. punctifer* reared at 28 °C and in complete darkness. Data are expressed as means ± standard deviation (S.D.) (*n* = 45). Different superscript letters indicate statistically significant differences between dietary treatments (one-way ANOVA, *p* < 0.05). Dietary treatment codes correspond to the protein:lipid level included in the tested diets.

Parameters	Dietary Treatments			
	30:15	30:10	45:15	45:10
Total length (mm)				
12 dpf	11.92 ± 0.50	12.17 ± 0.47	12.19 ± 0.54	11.90 ± 0.70
20 dpf	22.45 ± 0.41 ^b^	20.54 ± 1.08 ^b^	25.78 ± 1.24 ^a^	21.09 ± 0.94 ^b^
26 dpf	38.35 ± 1.48 ^b^	33.02 ± 0.77 ^c^	46.90 ± 0.98 ^a^	37.03 ± 0.94 ^b^
Wet weight (mg)				
12 dpf	7.32 ± 0.74	7.19 ± 0.20	8.09 ± 0.35	7.49 ± 1.71
20 dpf	68.04 ± 4.90 ^b^	55.31 ± 7.97 ^b^	97.91 ± 19.48 ^a^	60.63 ± 0.86 ^b^
26 dpf	264.44 ± 19.80 ^c^	220.85 ± 8.40 ^d^	563.71 ± 8.75 ^a^	306.76 ± 11.87 ^b^
Survival (%)				
12 dpf	93.88 ± 6.77	85.62 ± 7.14	78.58 ± 5.88	85.21 ± 9.10
26 dpf	17.68 ± 1.52 ^c^	12.66 ± 2.29 ^d^	36.32 ± 1.23 ^a^	23.44 ± 0.59 ^b^

**Table 3:** Composition of the experimental diets after manufacture. Data are expressed as mean *±* S.D. (*n* = 3). Different superscript letters denote the presence of differences statistically significant between dietary treatments (one-way ANOVA, *p* < 0.05). Dietary treatment codes correspond to the protein:lipid level included in tested diets. DM, dry matter.

Analyses of the Diets (% DM)	Dietary Treatments			
	30:15	30:10	45:15	45:10
Proteins	30.07 ± 2.44 ^b^	30.90 ± 4.01 ^b^	43.13 ± 2.65 ^a^	42.86 ± 2.03 ^a^
Lipids	12.50 ± 1.21 ^a^	7.43 ± 0.27 ^b^	12.46 ± 0.51 ^a^	10.36 ± 0.69 ^b^
Carbohydrates	24.87 ± 1.22 ^b^	31.31 ± 2.26 ^a^	2.34 ± 0.17 ^d^	7.58 ± 0.60 ^c^
Moisture	18.89 ± 0.16 ^b^	22.35 ± 0.02 ^a^	17.48 ± 0.04 ^c^	15.25 ± 0.07 ^d^
Energy (KJ)	1387.50 ± 24.89	1316.13 ± 43.67	1203.68 ± 81.53	1291.56 ± 54.47
E:P ratio (kcal g^−1^ protein)	11.07 ± 1.10 ^a^	10.19 ± 0.73 ^a^	6.66 ± 0.04 ^b^	7.20 ± 0.04 ^b^

**Table 4:** Proximate composition (in % of dry matter) of *P. punctifer* individuals (26 dpf) fed the different diets. Data are expressed as mean *±* S.D. (*n* = 3). Different superscript letters denote the presence of differences statistically significant between dietary treatments (one-way ANOVA, *p* < 0.05). Dietary treatment codes correspond to the protein:lipid level included in the experimental diets.

Proximate Composition (%)	Dietary Treatments			
	30:15	30:10	45:15	45:10
Proteins	52.70 ± 4.97	52.78 ± 1.79	47.06 ± 3.76	56.55 ± 3.65
Lipids	13.21 ± 0.23 ^a^	13.02 ± 0.87 ^a^	9.07 ± 0.45 ^b^	11.21 ± 0.59 ^a^
Carbohydrates	5.72 ± 0.02 ^a^	5.95 ± 0.22 ^a^	1.66 ± 0.12 ^c^	3.31 ± 0.33 ^b^
Ashes	1.65 ± 0.01	1.38 ± 0.03	1.80 ± 0.37	1.47 ± 0.09

**Table 5:** Total lipid and total fatty acids contents (in % of dry matter) and fatty acid composition (in % TFA) analyzed in the experimental diets. Data are expressed as mean *±* S.D. (*n* = 3). Different superscript letters denote the presence of differences statistically significant between dietary treatments (one-way ANOVA, *p* < 0.05). Dietary treatment codes correspond to the protein:lipid level included in the tested diets.

Total Lipids and Fatty Acids	Dietary Treatments			
	30:15	30:10	45:15	45:10
Total lipid (mg g^−1^ DW)	124.99 ± 12.07 ^a^	74.31 ± 2.75 ^b^	124.58 ± 5.07 ^a^	103.62 ± 11.64 ^ab^
Total fatty acid (mg g^−1^ DW)	71.18 ± 7.85 ^a^	38.91 ± 6.76 ^b^	66.81 ± 3.38 ^a^	52.16 ± 2.50 ^ab^
14:0	0.82 ± 0.04 ^c^	1.72 ± 0.00 ^a^	1.04 ± 0.00 ^c^	1.55 ± 0.25^ab^
16:0	19.71 ± 1.86	20.99 ± 1.54	19.21 ± 1.21	19.94 ± 1.48
18:0	3.62 ± 0.52	3.37 ± 0.33	3.09 ± 0.18	3.15 ± 0.40
Total saturated	24.15 ± 1.30	26.08 ± 1.21	23.34 ± 1.02	24.64 ± 1.33
16:1	1.72 ± 0.79	3.60 ± 1.74	2.26 ± 0.90	3.09 ± 1.26
18:1n-9 (OA)	19.15 ± 2.90	16.71 ± 0.99	19.41 ± 1.26	19.16 ± 0.70
20:1	2.78 ± 0.04 ^c^	6.61 ± 0.45 ^a^	4.42 ± 0.46 ^b^	7.06 ± 0.62 ^a^
Total monounsaturated	23.66 ± 2.1 6 ^b^	26.91 ± 0.30 ^ab^	26.09 ± 0.82 ^ab^	29.31 ± 0.06 ^a^
18:2n-6 (LA)	33.88 ± 0.38 ^a^	2.33 ± 0.17 ^c^	26.99 ± 0.99 ^b^	2.69 ± 0.46 ^c^
18:3n-6	0.00 ± 0.00	0.21 ± 0.08	0.00 ± 0.00	0.08 ± 0.11
20:4n-6 (ARA)	0.16 ± 0.22	1.10 ± 0.35	0.16 ± 0.22	0.26 ± 0.37
22:5n-6	0.00 ± 0.00	0.10 ± 0.15	0.00 ± 0.00	0.00 ± 0.00
Total n-6 PUFA	34.03 ± 0.60 ^a^	3.75 ± 0.45 ^c^	27.15 ± 1.21 ^b^	3.02 ± 0.02 ^c^
18:3n-3 (LLA)	2.80 ± 0.37 ^a^	0.73 ± 0.02 ^b^	2.39 ± 0.16 ^a^	0.83 ± 0.06 ^b^
18:4n-3	0.29 ± 0.28	0.00 ± 0.00	0.31 ± 0.44	0.44 ± 0.62
20:4n-3	0.13 ± 0.18	0.30 ± 0.01	0.25 ± 0.05	0.37 ± 0.01
20:5n-3 (EPA)	6.11 ± 0.34 ^c^	16.44 ± 0.21 ^a^	9.12 ± 0.62 ^b^	16.82 ± 0.69 ^a^
21:5n-3	0.00 ± 0.00	0.06 ± 0.09	0.05 ± 0.06	0.14 ± 0.01
22:5n-3 (DPA)	0.30 ± 0.16	0.81 ± 0.20	0.48 ± 0.11	0.87 ± 0.24
22:6n-3 (DHA)	7.98 ± 0.20 ^b^	24.00 ± 0.91 ^a^	10.36 ± 0.98 ^b^	22.43 ± 1.14 ^a^
Total n-3 PUFA	17.62 ± 0.45 ^c^	42.34 ± 0.59 ^a^	22.96 ± 0.91 ^b^	41.91 ± 0.92 ^a^
Total PUFA	51.65 ± 1.04 ^a^	46.09 ± 1.04 ^b^	50.11 ± 0.31 ^a^	44.93 ± 0.90 ^b^
(n-3)/(n-6)	0.52 ± 0.00 ^c^	11.37 ± 1.21 ^b^	0.85 ± 0.07^c^	13.86 ± 0.41^a^
DHA/EPA	1.31 ± 0.04 ^ab^	1.46 ± 0.07 ^a^	1.14 ± 0.03 ^b^	1.33 ± 0.01 ^ab^
ARA/DHA	0.02 ± 0.03	0.05 ± 0.01	0.02 ± 0.02	0.01 ± 0.02
ARA/EPA	0.03 ± 0.04	0.07 ± 0.02	0.02 ± 0.03	0.02 ± 0.02
LA/PUFA	0.66 ± 0.01 ^a^	0.05 ± 0.00^c^	0.54 ± 0.02 ^b^	0.06 ± 0.01 ^c^
LLA/PUFA	0.05 ± 0.01 ^a^	0.02 ± 0.00^b^	0.05 ± 0.00 ^a^	0.02 ± 0.00 ^b^
OA/PUFA	0.37 ± 0.06	0.36 ± 0.01	0.39 ± 0.03	0.43 ± 0.01
PUFA/saturated	2.14 ± 0.07	1.77 ± 0.12	2.15 ± 0.08	1.83 ± 0.14

**Table 6:** Total lipid and total fatty acids contents (in % of dry matter) and fatty acid composition (in % TFA) analyzed in *P. punctifer* individuals of 26 dpf. Data are expressed as mean *±* S.D. (*n* = 3). Different superscript letters denote differences statistically significant between dietary treatments (one-way ANOVA, *p* < 0.05). Dietary treatment codes correspond to the protein:lipid level included in the tested diets. TFA, total fatty acids.

Total Lipids and Fatty Acids	Dietary Treatments			
	30:15	30:10	45:15	45:10
Total lipid (mg g^−1^ DW)	132.11 ± 2.34 ^a^	130.23 ± 8.69 ^a^	90.74 ± 4.51 ^b^	112.07 ± 5.94 ^a^
Total fatty acid (mg g^−1^ DW)	88.99 ± 4.62 ^a^	79.86 ± 2.03 ^a^	59.33 ± 3.15 ^b^	65.94 ± 2.40 ^ab^
14:0	0.47 ± 0.07 ^b^	1.07 ± 0.07 ^a b^	0.70 ± 0.13 ^ab^	1.10 ± 0.21 ^a^
16:0	23.34 ± 0.41	23.96 ± 0.78	23.09 ± 0.37	23.83 ± 1.73
18:0	8.06 ± 0.04	8.60 ± 0.36	7.79 ± 0.63	8.28 ± 0.63
Total saturated	31.88 ± 0.53	33.63 ± 1.24	31.58 ± 0.38	33.21 ± 2.61
16:1	1.49 ± 0.12 ^b^	1.92 ± 0.09 ^a^	1.47 ± 0.15 ^b^	1.89 ± 0.09 ^a^
18:1n-9 (OA)	14.21 ± 0.21	13.75 ± 0.10	14.36 ± 0.14	13.80 ± 0.30
20:1	4.27 ± 0.10	4.92 ± 0.51	4.00 ± 0.25	5.00 ± 0.33
Total monounsaturated	19.98 ± 0.15	20.59 ± 0.48	19.83 ± 0.22	20.69 ± 0.83
18:2n-6 (LA)	9.86 ± 0.38 ^b^	3.10 ± 0.28 ^c^	12.81 ± 0.93 ^a^	3.00 ± 0.45 ^c^
18:3n-6	0.00 ± 0.00	0.00 ± 0.00	0.00 ± 0.00	0.00 ± 0.00
20:4n-6 (ARA)	1.35 ± 0.05	1.37 ± 0.04	1.25 ± 0.05	1.43 ± 0.12
22:5n-6	0.27 ± 0.01	0.26 ± 0.04	0.25 ± 0.03	0.28 ± 0.04
Total n-6 PUFA	11.48 ± 0.34 ^b^	4.72 ± 0.32 ^c^	14.31 ± 0.94 ^a^	4.79 ± 0.31 ^c^
18:3n-3 (LLA)	0.99 ± 0.12 ^a^	0.70 ± 0.02 ^b^	1.19 ± 0.12 ^a^	0.68 ± 0.07 ^b^
18:4n-3	0.59 ± 0.04 ^b^	0.70 ± 0.05 ^a^	0.56 ± 0.01 ^b^	0.69 ± 0.02 ^a^
20:4n-3	0.57 ± 0.00 ^b^	0.67 ± 0.00 ^a^	0.55 ± 0.00 ^b^	0.67 ± 0.02 ^a^
20:5n-3 (EPA)	8.44 ± 0.14 ^b^	9.88 ± 0.14 ^a b^	7.57 ± 0.11 ^b^	10.25 ± 0.73 ^a^
21:5n-3	0.29 ± 0.00	0.51 ± 0.02	0.27 ± 0.00	0.45 ± 0.21
22:5n-3 (DPA)	2.08 ± 0.04 ^a b^	2.27 ± 0.09 ^a^	1.90 ± 0.06 ^b^	2.37 ± 0.34 ^a^
22:6n-3 (DHA)	18.97 ± 0.06 ^b^	21.19 ± 0.43 ^ab^	17.22 ± 1.05 ^b^	21.57 ± 0.98 ^a^
Total n-3 PUFA	31.94 ± 0.33 ^b^	35.92 ± 0.59 ^a^	29.25 ± 1.02 ^b^	36.68 ± 1.71 ^a^
Total PUFA	43.42 ± 0.01 ^ab^	40.64 ± 0.26 ^b^	43.89 ± 0.68 ^a^	41.47 ± 1.56 ^b^
(n-3)/(n-6)	0.56 ± 0.02 ^c^	7.61 ± 0.27 ^a^	1.28 ± 0.11 ^b^	7.68 ± 0.74 ^a^
DHA/EPA	2.53 ± 0.08 ^a^	2.50 ± 0.01 ^a^	2.52 ± 0.15 ^a^	2.11 ± 0.07 ^b^
ARA/DHA	0.10 ± 0.01 ^a^	0.06 ± 0.00 ^b^	0.07 ± 0.00 ^b^	0.07 ± 0.00 ^b^
ARA/EPA	0.25 ± 0.02 ^a^	0.15 ± 0.01 ^b^	0.19 ± 0.01 ^a b^	0.14 ± 0.00 ^b^
LA/PUFA	0.60 ± 0.01 ^a^	0.14 ± 0.01 ^c^	0.40 ± 0.02 ^b^	0.07 ± 0.01^d^
LLA/PUFA	0.04 ± 0.00 ^a^	0.02 ± 0.00 ^c^	0.03 ± 0.00 ^b^	0.02 ± 0.00 ^c^
OA/PUFA	0.33 ± 0.00	0.31 ± 0.00	0.32 ± 0.01	0.33 ± 0.01
PUFA/saturated	1.43 ± 0.02	1.25 ± 0.05	1.40 ± 0.04	1.24 ± 0.14

**Table 7:** Hepatocyte number in the hepatic parenchyma and surface (S) of lipid vacuoles within hepatocytes of *P. punctifer* specimens (26 dpf) fed diets containing different protein:lipid levels (30:15, 30:10, 45:15, and 45:10). Data represent means ± S.D. of three replicate tests for each dietary treatment (*n* = 5 fish per replicate). Different letters within column denote statistically significant differences among dietary groups (one-way ANOVA, *p* < 0.05).

Dietary Treatments	Hepatocyte Number (in 100 µm^2^)	S Hepatic Lipid Vacuoles (µm^2^)
30:15	30 ± 0.9 ^b^	140.99 ± 15.68 ^a^
30:10	19 ± 1.2 ^c^	117.89 ± 4.35 ^a^
45:15	54 ± 3.2 ^a^	19.97 ± 1.81 ^c^
45:10	28 ± 2.1 ^b^	26.53 ± 2.34 ^b^

**Table 8:** Number and size of different components of the intestinal mucosa of *P. punctifer* specimens (26 dpf) fed the four different dietary treatments containing different protein:lipid levels (in % of dry matter). Data represent means ± S.D. of three replicate tanks for each dietary treatment (*n* = 5 fish per replicate). Different letters from “a” to “c” within columns denote statistically significant differences among dietary treatments within the same intestinal section (one-way ANOVA, *p* < 0.05). Different letters from “x” to “z” denote statistically significant differences between intestinal sections within the same dietary treatment (one-way ANOVA, *p* < 0.05).

Dietary Treatments	N° Enterocytes (in 100 µm)	Height Enterocytes (µm)	N° Folds (in 100 µm^2^)	Length Folds (µm)	N° Lipid Droplets (in 100 µm)	S. Lipid Droplets (µm^2^)	N° Goblet Cells (in 100 µm)
Anterior intestine							
30:15	30.0 ± 1.4 ^ax^	16.9 ± 1.2 ^by^	1.0 ± 0.0 ^ay^	154.8 ± 122.6	0.0 ± 0.0 ^y^	0.0 ± 0.0	3.8 ± 1.5 ^b^
30:10	20.7 ± 1.2 ^bx^	15.6 ± 1.2 ^by^	0.0 ± 0.0 ^b^	0.0 ± 0.0 ^y^	0.0 ± 0.0 ^y^	0.0 ± 0.0	3.9 ± 0.9 ^bx^
45:15	26.0 ± 2.0 ^ax^	14.4 ± 0.9 ^bz^	1.0 ± 0.0 ^a^	151.7 ± 54.9 ^x^	0.0 ± 0.0 ^y^	0.0 ± 0.0 ^y^	11.0 ± 2.0 ^ax^
45:10	18.0 ± 2.0 ^bx^	22.4 ± 1.6^ax^	1.0 ± 0.0 ^ay^	282.8 ± 148.1	0.0 ± 0.0 ^y^	0.0 ± 0.0 ^y^	1.0 ± 0.9 ^by^
Middle intestine							
30:15	22.3 ± 1.5 ^ay^	23.0 ± 4.1 ^axy^	2.2 ± 0.4 ^bx^	75.4 ± 21.3 ^a^	0.0 ± 0.0 ^y^	0.0 ± 0.0	3.4 ± 1.9
30:10	16.3 ± 1.5 ^by^	19.6 ± 1.5 ^ax^	3.8 ± 0.3 ^a^	10.9 ± 15.4 ^cy^	0.0 ± 0.0 ^y^	0.0 ± 0.0	5.3 ± 0.9 ^x^
45:15	22.3 ± 1.5 ^ay^	19.8 ± 0.9 ^ay^	1.3 ± 0.6 ^b^	37.3 ± 0.1 ^bcy^	0.0 ± 0.0 ^y^	0.0 ± 0.0 ^y^	3.3 ± 1.3 ^y^
45:10	18.0 ± 1.4 ^bx^	11.5 ± 0.8 ^bz^	2.0 ± 0.0 ^bx^	56.71 ± 0.9 ^ab^	0.0 ± 0.0 ^y^	0.0 ± 0.0 ^y^	4.1 ± 1.4 ^x^
Posterior intestine							
30:15	11.3 ± 1.5 ^bz^	27.8 ± 5.3 ^bx^	0.7 ± 0.3 ^y^	166.21 ± 9.1 ^ab^	7.5 ± 1.9 ^bx^	148.6 ± 107.5	3.0 ± 1.6
30:10	13.0 ± 0.7 ^bz^	19.7 ± 0.5 ^cx^	0.5 ± 0.7	111.5 ± 27.7 ^bx^	7.0 ± 1.1 ^bx^	48.4 ± 68.4	1.2 ± 0.5 ^y^
45:15	18.3 ± 0.5 ^az^	37.8 ± 4.5 ^ax^	1.0 ± 0.0	227.9 ± 40.0 ^ax^	11.5 ± 0.5 ^ax^	301.5 ± 122.5 ^x^	1.8 ± 1.3 ^y^
45:10	11.7 ± 0.6 ^by^	19.6 ± 1.1 ^bcy^	0.7 ± 0.6 ^y^	216.6 ± 52.3 ^ax^	11.3 ± 1.2 ^ax^	362.4 ± 141.4 ^x^	0.3 ± 0.5 ^y^

## Data Availability

Data presented in this study are available from the corresponding authors upon reasonable request.
